# Impact of Observation Duration in Action Observation Therapy: Manual Dexterity, Mirror Neuron System Activity, and Subjective Psychomotor Effort in Healthy Adults

**DOI:** 10.3390/brainsci15050457

**Published:** 2025-04-27

**Authors:** Anri Sasaki, Eizaburo Suzuki, Kotaro Homma, Nariyuki Mura, Katsuhiko Suzuki

**Affiliations:** 1 Department of Rehabilitation, Ishinomaki Loyal Hospital, Ishinomaki 987-1222, Japan; a.takahashi929@outlook.jp; 2 Department of Physical Therapy, Yamagata Prefectural University of Health Sciences, Yamagata 990-2212, Japan; nmura@yachts.ac.jp (N.M.); ksuzuki@yachts.ac.jp (K.S.); 3 Department of Rehabilitation, Sonoda Third Hospital, Tokyo 121-0807, Japan; latiss.k24@gmail.com; 4 Department of Rehabilitation, Sonoda a Medical Institute Tokyo Spine Center, Tokyo 121-0807, Japan

**Keywords:** observation duration, action observation therapy, mirror neuron system, manual dexterity, interpersonal motor resonance, motor-evoked potential, intermanual transfer, psychomotor effort, concentration, physical fatigue, mental fatigue

## Abstract

**Background/Objectives:** Action observation therapy (AOT) has gained attention as a rehabilitation method for motor function recovery following nerve injury. Although the total observation time and daily session duration have been studied, the effective observation duration per trial remains unclear. This study examined the effect of different observation durations on manual dexterity, mirror neuron system activity, and subjective psychomotor effort in healthy adults. **Methods:** Twenty-four healthy right-handed adults participated in this crossover study under four conditions: observing ball rotations with the dominant hand for one, two, or three minutes, or geometric patterns (control) for two minutes. The outcomes included maximum rotations and errors by both hands during a ball rotation task and interpersonal motor resonance (IMR), indicating mirror neuron system activity. These measures were compared before and after intervention. Subjective ratings of concentration, physical fatigue, and mental fatigue were assessed post-intervention. **Results:** Rotation performance significantly increased for the intervention hand after a 2 min observation and showed a notable effect (*p* = 0.113, r = 0.48) for the non-intervention hand after a 3 min observation compared to the control. The IMR was significantly greater during the 2 min observation than in the control. Compared to the 1 min observation, the 2 min and 3 min observations resulted in higher mental fatigue, and the 3 min observation showed lower concentration levels. **Conclusions:** These findings indicate that the observation duration has varying effects on manual dexterity and mirror neuron system activity, with optimal effects observed at specific time intervals while also highlighting the relationship between observational learning and psychomotor effort.

## 1. Introduction

Action observation therapy (AOT) is a rehabilitation approach that aims to improve motor function recovery through repeated cycles of observation and execution of movements. This technique has shown particular promise in patients with upper limb paralysis resulting from stroke [1,2,3,4,5,6]. The neurophysiological basis of AOT lies in its ability to activate brain regions associated with damaged motor areas, even in patients with moderate to severe motor paralysis who have difficulty with voluntary movement or muscle contraction [7,8,9]. Furthermore, AOT functions as a multisensory integrative approach, engaging not only motor systems, but also visual and cognitive functions, thereby potentially enhancing motor learning through imitation [10]. Despite these theoretical advantages and the broad applicability of AOT across various patient populations, the clinical evidence supporting its effectiveness remains incomplete. Recent systematic reviews have reported varying effects: relatively large improvements in hand function but only small effects on upper-extremity motor function [11]. Moreover, these reviews have identified two significant limitations in the current evidence base: first, the observed improvements may not reach the threshold for clinically meaningful differences, and second, the overall certainty of the available evidence has been rated as low [7]. These limitations highlight the need for further investigation into the efficacy and optimal application of AOT.

The mechanism underlying the effects of AOT on motor function is primarily attributed to the mirror neuron system (MNS), a fundamental neural substrate for motor observation [12]. Mirror neurons (MNs) are specialized visual-motor neurons activated when observing and performing actions [13]. Initially discovered in the ventral premotor area of macaque monkeys [14] and later in the inferior parietal lobule [15], homologous MNS regions in humans include the ventral premotor cortex, inferior parietal lobule, and inferior frontal gyrus [13]. Research has demonstrated that movement observation activates the frontoparietal network, which subsequently engages motor-related brain areas during movement execution [12,16,17]. Studies from a motor learning perspective have shown that MNS regions are activated during the early stages of skill acquisition [18], whereas the neural bases of observational learning substantially overlap with those of physical practice [19]. These findings suggest that AOT’s efficacy stems from its combination of movement observation and execution, engaging both the MNS and motor execution areas, thereby facilitating the formation of appropriate motor programs in the premotor cortex and parietal regions [12].

Although there are certain beliefs regarding the mechanisms of AOT, there is a lack of knowledge regarding the extent of AOT interventions necessary to improve motor function [2]. Furthermore, there are no standardized procedures detailing when and how AOT should be implemented, including aspects such as the optimal dosage and intervention protocols. A recent meta-analysis recommended that treatment should begin 23 days or later after stroke onset, with 30–40 min sessions 3–5 times a week for at least 4 weeks [20]. In a subsequent sub-analysis of this study, the effects of AOT on arm function were analyzed by observation duration per trial, and the results showed that observation durations longer than 3 min were more effective than times shorter than 3 min. However, further research is needed to compare the observation duration of these single trials, noting that the complexity and type of behavior or task being observed, and whether or not imitation is subsequently performed, varies widely.

Among these parameters, observation duration is critical for two important reasons. First, sustained visual attention during observation requires significant concentration, which can lead to mental fatigue and potentially diminish the therapeutic effects. Second, the amount of visual information processed during observations directly influences the degree of neural plasticity induced in the MNS and motor-related areas. Despite these considerations, no study has specifically investigated the effects of different observation durations during a single intervention session, particularly regarding hand function, and no experimental studies have compared interventions while controlling task type and complexity. Furthermore, the relationship between observation duration and changes in manual dexterity, MNS activity, and subjective psychomotor effort remains unclear. Therefore, this study investigated the impact of different observation durations (in minutes) on manual dexterity, MNS activity, and subjective psychomotor effort in healthy adults.

Given the lack of consistent evidence regarding the optimal observation duration for hand function in AOT, this study adopted an exploratory approach to investigate the effects of different observation durations (one, two, and three minutes) on manual dexterity, mirror neuron system activity, and subjective psychomotor effort. Rather than testing specific directional hypotheses, we aimed to identify potential relationships between observation duration and various outcomes, including behavioral performance (manual dexterity), neurophysiological responses (mirror neuron system activity), and subjective experience (psychomotor effort), which may inform future hypothesis-driven research and clinical applications.

This study provides practical knowledge regarding the optimal observation duration for AOT while also offering a multifaceted analysis of neurophysiological changes in MNS activity, behavioral changes in manual dexterity, and psychological factors during AOT interventions. These findings provide important insights into both the clinical application and theoretical understanding of AOT mechanisms.

## 2. Materials and Methods

### 2.1. Participants

Twenty-four healthy, right-hand-dominant adults aged between 18 and 50 years (mean age, 19 ± 1.24 years; 14 female participants) were included in this study. Handedness was assessed by using the Edinburgh Handedness Inventory [21]. The sample size required to achieve an effect size of 0.25, significance level (α) of 0.05, and power (1 − β) of 0.8 was calculated using the statistical power analysis software G*Power Version 3.1.9.6 [22]. All participants had adequate or corrected visual acuity; therefore, interference with the experiment related to eyesight was avoided. Only right-hand-dominant individuals were included to eliminate the influence of differences caused by handedness, because previous studies have found that MNS activity differs between right-hand-dominant and left-hand-dominant individuals [23,24,25]. The exclusion criteria were as follows: history of central nervous system disease; metal (postsurgical remains) or electronic devices (cardiac pacemaker) implanted in the brain, skull, or body; head trauma with impaired consciousness; history of epileptic seizures/convulsions; history of syncope; pregnancy or possible pregnancy; and current use of drugs affecting the central nervous system. These criteria were defined according to the International Society of Clinical Neurophysiology guidelines for the safety of transcranial magnetic stimulation methods [26].

All participants provided written informed consent after receiving verbal information regarding the study. This study was approved by the ethics committee of the Yamagata Prefectural University of Health Sciences (approval number: 2112-32) and conducted in accordance with the ethical standards of the Declaration of Helsinki.

### 2.2. Experimental Procedures

**Figure 1:**
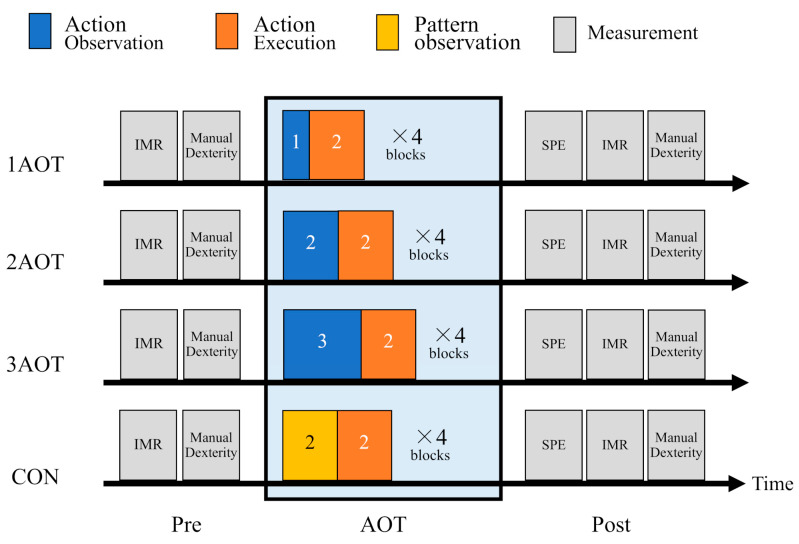
Experimental procedure. Action observation therapy (AOT) consisted of one block of action observations and execution and was repeated four times. Four AOT intervention conditions were used: action observation for 1 min (1AOT); action observation for 2 min (2AOT); action observation for 3 min (3AOT); and observation of a still image of a geometric pattern for 2 min (control [CON]). IMR, which reflects the mirror neuron system activity and manual dexterity in the ball rotation task, was measured pre- and post-AOT intervention. After the AOT intervention, the subjective psychomotor effort (SPE) consisting of concentration, physical fatigue, and mental fatigue was assessed.

Figure 1 illustrates the experimental procedure used in this study, which was designed as a crossover trial. The participants were subjected to all the intervention conditions on separate days. The AOT intervention conditions were as follows: observation of movement for 1 min (1AOT), observation of movement for 2 min (2AOT), observation of movement for 3 min (3AOT), and a control condition (CON) during which a geometric pattern was observed. A washout period of at least two weeks was provided between each condition to avoid any effects on the results. The order of the four conditions was counterbalanced among the participants, and 24 order sequences were randomly assigned using a random number table. MNS activity and finger motor function were evaluated before and after the AOT. After the AOT, subjective psychomotor effort (concentration, physical fatigue, and mental fatigue) was evaluated.

### 2.3. Action Observation Therapy

The participants sat in a chair and assumed a comfortable posture with both forearms placed in front of them and pronated on a table. A liquid crystal monitor (EX-LDGC271TB TFT27 wide GigaCrysta; IO Data, Phoenix, AZ, USA) was placed in front of the participants at a distance of 1 m from the eyes.

**Figure 2:**
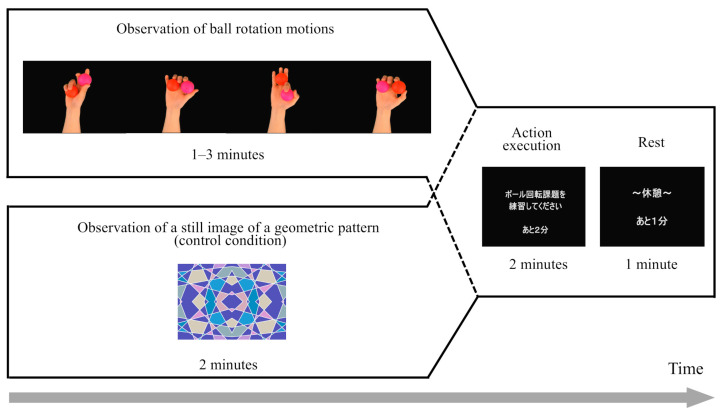
Images presented during action observation therapy. The motion observed and performed was ball rotation. During this task, two golf balls (diameter: 43 mm) rapidly rotated on the palm. A video of the balls rotating clockwise without touching each other using the right hand was shown during motion observation. The video observation times for each intervention were 1 min (1AOT condition), 2 min (2AOT condition), and 3 min (3AOT condition). However, in the control condition, participants observed a still image of a geometric pattern for 2 min instead of motion. After watching the video, participants performed the action execution for two minutes, followed by a one-minute break. During both the action execution and the break, the instructional Japanese text and the remaining time were displayed.

Figure 2 shows video images of the AOT interventions used in this study. The AOT is an intervention method that alternates between movement observation and execution. The observed and performed motions were ball rotation. During this task, two golf balls (diameter: 43 mm) rapidly rotated on the palm.

During the observation of motion, a video of the balls rotating clockwise without touching each other using the right hand was presented on the monitor. The rotation speed of the balls in the video was 15 min^−1^. Because interpersonal motor resonance (IMR) may be activated more when observing movements at a level similar to one’s own [22], this rotation speed was tested separately from this experiment among healthy young individuals who had not undergone any intervention. The rotational speed of the ball during the preliminary experiment was measured, and an average value was adopted. The participants were asked to carefully observe the ball rotation motion portrayed in the video; additionally, they were asked to observe the video without moving their fingers. Specifically, the following verbal instruction was given: “During the observation phase of the model video, please watch without moving your finger. Watch carefully so that you can apply it to the exercise execution phase.” The video observation times for the intervention conditions were 1 min (1AOT), 2 min (2AOT), and 3 min (3AOT). Additionally, as a control condition, the participants observed a still image of a geometric pattern for 2 min instead of motion.

During exercise execution, the participants practiced rotating the ball as quickly and accurately as possible with their right hand. Participants were specifically given the following instruction verbally: “In the exercise execution, please feel free to practice the ball rotation task with your right hand. If you can rotate faster than the model video, you may perform faster.” The exercise execution condition had a duration of 2 min. A 1 min break was provided after each exercise observation and execution condition, and four condition sessions were used. The start and end of the exercise observation, exercise execution, and rest were signaled by displaying numbers on the monitor and a beeping sound to indicate the countdown for each phase.

### 2.4. Evaluation of Manual Dexterity

The ball rotation task used during AOT was adopted for manual dexterity evaluation. Participants were instructed to hold two golf balls while sitting on a chair and to rotate the balls as quickly and accurately as possible within 20 s of the starting signal without allowing the balls to hit each other. The maximum number of rotations for the right and left hands were measured in five trials each, with the right hand rotated clockwise and the left hand rotated counterclockwise. Measurements of the right hand were performed to evaluate the direct practice effect of AOT, and measurements of the left hand were performed to verify whether learning through AOT with the right hand affected execution of the left hand. This generalization of learning to the untrained hand is called bimanual transfer and is associated with movement planning that does not depend on effector organs [27]. Previous studies [28,29] suggest that intermanual transfer occurs during both movement observation and execution. Therefore, AOT, which combines movement observation and execution, may also induce transfer between both hands; consequently, this was also evaluated in this study.

Rotational motion was recorded with a web camera (Logicool C930 PRO HD; Logitech, Lausanne, Switzerland), and both the number of ball rotations and errors were analyzed offline. An error was defined as contact between the golf balls or dropping one or both balls; if an error occurred, the trial was interrupted, and the next trial was measured. If an error occurred during all five trials, the maximum number of rotations was set to zero.

The median of the maximum rotation values from the five trials was calculated as the representative value for each individual. Additionally, to compare the intervention conditions, the median of the change in the maximum number of rotations or errors before and after the intervention (post-intervention to pre-intervention) was calculated.

### 2.5. Evaluation of Mirror Neuron System Activity

#### 2.5.1. Interpersonal Motor Resonance

IMR is a method used to evaluate MNS activity. IMR is a phenomenon in which an observer’s motor system is activated by observing the movements performed by others [30,31,32]. It can be quantitatively evaluated using an experimental paradigm based on TMS [33]. Generally, by administering a single magnetic stimulus to the primary motor cortex of the skull (scalp) using TMS, the motor-evoked potential (MEP) is measured in the contralateral skeletal muscle corresponding to the stimulation site. The peak-to-peak MEP amplitude reflects the excitability of the corticospinal tract [34]. When IMR occurs during motion observation, the MEP amplitude during motion observation increases compared to that during non-motion observation. This increase in MEP amplitude is thought to be a temporary increase in the excitability of the primary motor cortex caused by MNS activation, and indirectly reflects MNS [35]. Additionally, the IMR response has muscle specificity, and specific activation occurs in the muscles involved in execution of the observed movement [36]. During this study, the rate of increase in the MEP amplitude or IMR was used as an indicator of MNS activity.

#### 2.5.2. Measurement of Interpersonal Motor Resonance

The participants were placed in a comfortable sitting position, and their heads were fixed with their chins resting on a height-adjustable chin rest. Ag/AgCl surface electrodes (BlueSensor NF; Ambu, Ballerup, Denmark) were attached to the belly of the abductor pollicis brevis (APB) muscle and the abductor digiti minimi (ADM) muscle of the right hand at a distance of 10 mm between electrodes. The electromyography signal obtained by applying a low-pass filter up to 1 kHz was amplified using an amplifier (FE232 dual bioamplifier; ADInstruments, Colorado Springs, CO, USA), passed through an analog-to-digital converter (PowerLab 8/35, ADInstruments), and sent to a personal computer at a sampling frequency of 4 kHz (G7 17; Dell, Round Rock, TX, USA). Electromyogram analysis software (LabChart Pro v8 upgrade; ADInstruments) was used for analyses. The time window for the MEP amplitude measurement was 80 ms (from 20 ms before stimulation to 60 ms after stimulation). TMS was performed using a magnetic stimulator (Magstim 200 Stimulator; Magstim, Whitland, UK) with a double alpha coil (diameter, 70 mm).

When measuring the MEP using TMS, the optimal stimulation position (hotspot) on the left primary motor cortex, where the amplitude values of the MEPs of the APB and ADM of the participant were at maximum, was measured vertically in increments of 1 cm using the international 10–20 method. This was identified by the application of a stimulus. The resting motor threshold (RMT) at the hotspot was measured to determine the stimulus intensity. RMT is the minimum stimulus intensity at which an MEP amplitude of 50 μV or more can be induced with a 50% probability. According to previous studies, the stimulus intensity of TMS administered during observation of the video and still image was set at 110% of the RMT [37,38]. When the RMTs of the APB and ADM differed, a higher value was used as the criterion for stimulus intensity.

**Figure 3:**
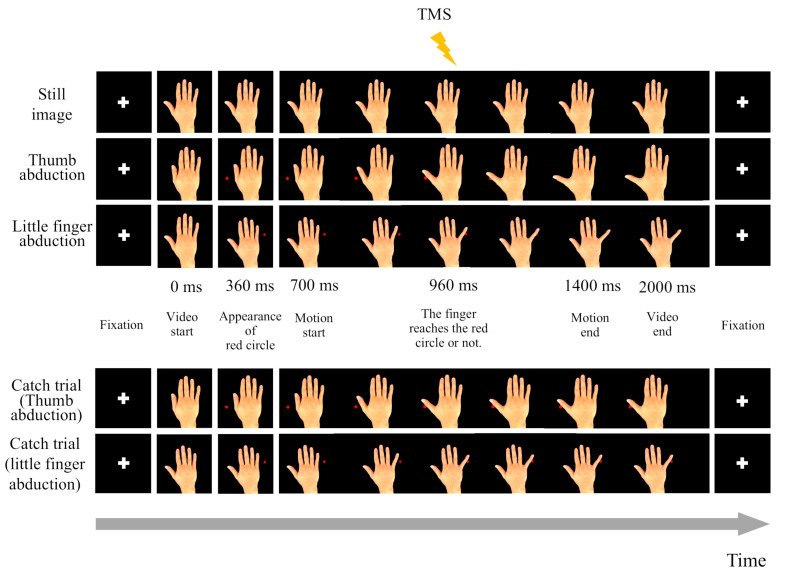
Interpersonal motor resonance measurements. Three types of visual stimuli, a still image of the right hand, abduction of the thumb, and abduction of the little finger, were presented individually for two seconds. For thumb or little finger abduction, a red circle (target) appeared 360 ms after the video started, and the finger began to overlap with the red circle after 700 ms. After 960 ms, the finger touched the red circle, at which point transcranial magnetic stimulation (TMS) stimulation was applied, and the abductor pollicis brevis (APB) and abductor digiti minimi (ADM) motor-evoked potentials (MEPs) were measured. The TMS stimulus was applied 960 ms after a still image was displayed. Before and after the three types of visual stimuli, a cross symbol was randomly presented for 2–3 s to fix the gaze and attention of the participants. A catch trial was randomly inserted only once during each trial. During the catch trial, the movement was stopped when the finger touched the red circle. Participants were asked whether there was a catch trial and which finger movements were performed immediately after the evaluation. The participants were asked to maintain their gaze and concentration.

Figure 3 shows the IMR measurement procedure. Three types of visual stimuli, a still image of the right hand, abduction of the thumb, and abduction of the little finger, were sequentially presented for 2 s on a liquid-crystal monitor placed in front of the participants. During the abduction movement of the thumb or little finger, a red circle (target) appeared in the direction of that movement 360 ms after the start of the video. The finger began to overlap the red circle after 700 ms. After 960 ms, the finger touched the red circle, TMS was administered, and the MEPs of the APB and ADM were measured. A single stimulus was provided during TMS, 960 ms after the still image was displayed. Each of the three types of visual stimuli was presented 13 times for a total of 39 times, and the same number of MEP data points were acquired. During this process, participants were asked to concentrate on observing the movement of their finger until it touched the red circle. Before and after the three types of visual stimuli were presented, a cross symbol was randomly presented for 2 to 3 s to fix the gaze and attention of the participants. Additionally, to maintain the concentration of the participants, we randomly inserted a trial (catch trial) during which the movement was stopped when the finger touched the red circle only once. The presentation of the catch trial and the finger movements were both random. Participants were asked whether there was a catch trial and which finger movements were performed immediately after the evaluation. Numerical analysis software (MATLAB R2020b; MathWorks Inc., Natick, MA, USA) was used to control the presentation of the video and the stimulation timing during TMS. The IMR was calculated using the following equation and the MEP amplitude values obtained during observations of the still image, thumb abduction, and little finger abduction [39]:

$$IMR\;=\;\frac{\left(MEP\;amplitude\;during\;movement\;observation\;-\;MEP\;ampitude\;during\;still\;imgae\;observation\right)}{MEP\;ampitude\;during\;still\;imgae\;observation}\;\times \;100$$

Of the 13 IMR measurements, values that exceeded three times the standard deviation were excluded from analysis. After exclusion, the average value was used as the representative value. Based on a previous study [40], the number of MEP measurements was set to 13 to analyze MEPs with at least 10 iterations (not including missing and excluded values). The evaluation was performed twice for each experimental condition (pre- and post-intervention).

### 2.6. Subjective Psychomotor Effort (Concentration, Physical Fatigue, and Mental Fatigue)

Immediately after AOT, the concentration, physical fatigue, and mental fatigue caused by AOT were evaluated using an 11-point numerical rating scale (NRS), with scores ranging from 0 to 10. These results are used as supplementary data. Higher concentration scores indicate better concentration. Higher fatigue scores indicate worse fatigue.

### 2.7. Statistical Analysis

Following the Shapiro–Wilk test for normality assessment, all data were subsequently analyzed using nonparametric statistical methods. The Wilcoxon signed-rank test was used to compare all variables before and after the intervention. Friedman’s test was performed to compare changes in all variables and NRS scores of subjective psychomotor effort between intervention conditions. The Bonferroni-corrected Wilcoxon signed-rank test was used for subsequent multiple comparisons.

The effect size (*r*) was calculated during the multiple comparison test of both the number of rotations and the number of errors during the ball rotation task, IMR, and NRS scores using the *Z*-value and number of participants. The effect size (*w*) for Friedman’s test was calculated using *χ*^2^, the number of participants, and the number of conditions. Based on the work by Cohen (1988) [41], *r* ≥ 0.5 was defined as large, *r* ≥ 0.3 was defined as medium, *r* ≥ 0.1 was defined as small, and *r* < 0.1 was defined as almost none. For Kendall’s W, *w* = 0.5 was defined as large, *w* = 0.3 was defined as medium, and *w* = 0.1 was defined as small.

SPSS Statistics 28 (IBM, Armonk, NY, USA) was used to perform statistical analyses. All statistical tests were set at *p* < 5%.

## 3. Results

### 3.1. Performance of Ball Rotation Task

#### 3.1.1. The Maximum Number of Rotations in 20 s

**Figure 4:**
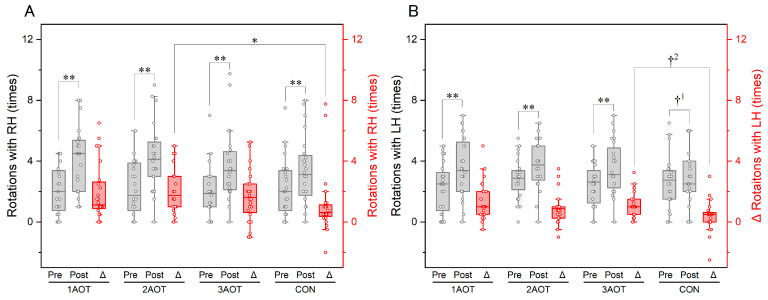
Maximum number of rotations in 20 s in the ball rotation task. (**A**) Maximum number of rotations with the right hand (intervention hand). (**B**) Maximum number of rotations with the left hand (non-intervention hand). RH, right hand; LH, left hand; AOT, action observation therapy; 1AOT, one-minute observation duration of AOT; 2AOT, two-minute observation duration of AOT; 3AOT, three-minute observation duration of AOT; CON, control condition (two-minute observation of geometric pattern); Pre, pre-intervention; Post, post-intervention; Δ, change in the maximum number of rotations (Post-Pre). *, Bonferroni adjusted *p* < 0.05; **, *p* < 0.01; †^1^, *p* = 0.052, *r* = 0.40; †^2^, *p* = 0.113, *r* = 0.48.

Regarding the right hand, the number of rotations during all conditions increased significantly after the intervention (Figure 4A, 1AOT: *p* < 0.01, *r* = 0.84; 2AOT: *p* < 0.01, *r* = 0.86; 3AOT: *p* < 0.01, *r* = 0.75; CON: *p* < 0.01, *r* = 0.68). Friedman’s test showed a significant difference in the change in the maximum number of rotations between the intervention conditions for the right hand [*χ*^2^(3) = 9.267, *p* = 0.026, *w* = 0.13]. Subsequent multiple comparisons showed that the change in the maximum number of rotations under the 2AOT condition was significantly larger than that under the control condition (adjusted *p* = 0.022; *r* = 0.59). With regard to the left hand, a significant increase was also observed after the intervention under the 1AOT, 2AOT, and 3AOT conditions (Figure 4B, 1AOT: *p* < 0.01, *r* = 0.82; 2AOT: *p* < 0.01, *r* = 0.71; 3AOT: *p* < 0.01, *r* = 0.84) but did not reach significance under the control condition, despite showing a medium to high effect size (CON: *p* = 0.052, *r* = 0.40). There was a significant difference in the change between intervention conditions [*χ*^2^(3) = 9.133, *p* = 0.028, *w* = 0.13], and subsequent multiple comparisons showed that the change in rotations under the 3AOT condition tended to be larger than that under the control condition (adjusted *p* = 0.113; *r* = 0.48), with a moderate effect size. A table showing the median, first quartile, third quartile, 95% confidence interval, test statistic (*χ*^2^(3) or *Z*), *p*-value, and effect size in all data comparisons is provided in Table S1.

#### 3.1.2. The Number of Errors

Using the right hand, the number of errors under the 1AOT, 3AOT, and control conditions decreased significantly after the intervention (1AOT: *p* < 0.01, *r* = 0.57; 3AOT: *p* < 0.01, *r* = 0.59; CON: *p* < 0.01, *r* = 0.56), and no significant change was observed under the 2AOT condition (*p* = 0.192; *r* = 0.27). However, Friedman’s test showed no significant difference in the changes in the number of errors between the intervention conditions for the right hand [*χ*^2^(3) = 2.95, *p* = 0.400]. Using the left hand, no significant change was observed under any condition after the intervention (1AOT, *p* = 0.768, *r* = 0.06; 2AOT, *p* = 0.949, *r* = 0.01; 3AOT, *p* = 0.461, *r* = 0.01; CON: *p* = 0.841, *r* = 0.05). Friedman’s test showed no significant difference in the changes in the number of errors between the intervention conditions for the left hand [*χ*^2^(3) = 5.07; *p* = 0.167]. A table showing the median, first quartile, third quartile, 95% confidence interval, test statistic (*χ*^2^(3) or *Z*), *p*-value, and effect size in all data comparisons is provided in Table S2.

### 3.2. Interpersonal Motor Resonance (Mirror Neuron System Activity)

**Figure 5:**
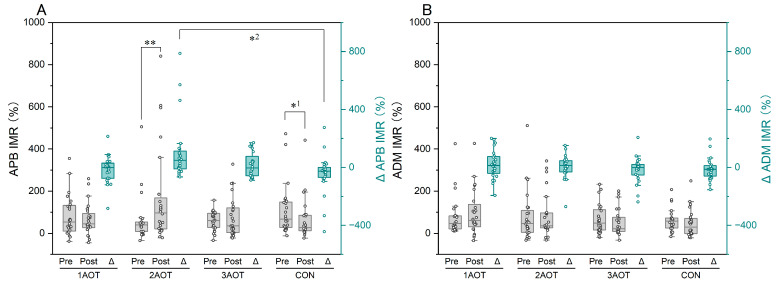
Interpersonal motor resonance. (**A**) IMR of APB. (**B**) IMR of ADM. AOT, action observation therapy; 1AOT, one-minute observation duration of AOT; 2AOT, two-minute observation duration of AOT; 3AOT, three-minute observation duration of AOT; CON, control condition (two-minute observation of geometric pattern); Pre, pre-intervention; Post, post-intervention; Δ, change in the maximum number of rotation (Post–Pre). *^1^, *p* < 0.05; **, *p* < 0.01; *^2^, Bonferroni adjusted *p* < 0.05.

Regarding the APB, the IMR in the 2AOT condition increased significantly after the intervention (Figure 5A, *p* < 0.01, *r* = 0.54), whereas it decreased significantly in the control condition (*p* < 0.05, *r* = 0.46). Friedman’s test showed a significant difference in the change in the IMR between the intervention conditions for the APB [*χ*^2^(3) = 8.150, *p* = 0.043, *w* = 0.13]. Subsequent multiple comparisons showed that the change in IMR under the 2AOT condition was significantly larger than that under the control condition (adjusted *p* = 0.031; *r* = 0.57). Regarding the ADM, no statistically significant effects were observed in the comparisons between pre- and post-intervention measurements or in the comparisons of change amounts between the conditions (Figure 5B). A table showing the median, first quartile, third quartile, 95% confidence interval, test statistic (*χ*^2^(3) or *Z*), *p*-value, and effect size in all data comparisons is provided in Table S3.

### 3.3. Subjective Psychomotor Effort (Concentration, Physical Fatigue, and Mental Fatigue)

**Figure 6:**
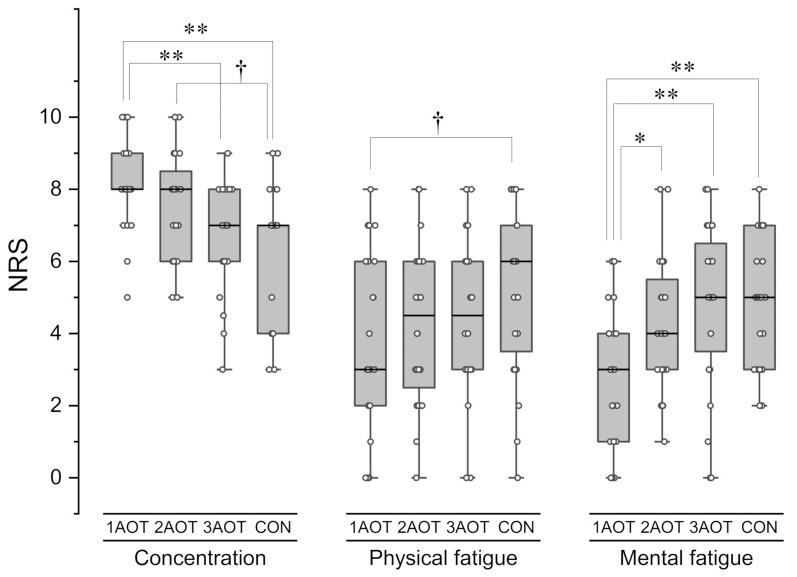
Subjective evaluation of concentration, physical fatigue, and mental fatigue after the intervention. Higher concentration scores indicated better concentration. Higher fatigue scores indicated worse fatigue. AOT, action observation therapy; 1AOT, one-minute observation duration of AOT; 2AOT, two-minute observation duration of AOT; 3AOT, three-minute observation duration of AOT; CON, control condition (two-minute observation of geometric pattern). *, *p* < 0.05; **, *p* < 0.01; †, *p* = 0.052, *r* = 0.54.

Regarding concentration, Friedman’s test showed a significant difference between the conditions [(*χ*^2^(3) = 26.472, *p* < 0.01, *w* = 0.37], and multiple comparisons showed that the NRS under the 1AOT condition was higher than those under the 3AOT and control conditions (Figure 6, 1AOT: adjusted *p* < 0.01, *r* = 0.70; CON: adjusted *p* < 0.01, *r* = 0.86), and that the NRS under the 2AOT condition tended to be higher than that under the control condition with a large effect size (adjusted *p* = 0.052, *r* = 0.54). Regarding physical fatigue, Friedman’s test showed a significant difference between the conditions [*χ*^2^(3) = 10.862, *p* = 0.012, *w* = 0.15]; subsequent multiple comparisons showed that the NRS under the 1AOT condition tended to be lower than that under the control condition, with a large effect size (adjusted *p* = 0.052, *r* = 0.54). Regarding mental fatigue, Friedman’s test showed a significant difference between the conditions [(*χ*^2^(3) = 26.803, *p* < 0.01, *w* = 0.37]; and subsequent multiple comparisons showed that the NRS under the 1AOT condition was lower than those under the 2AOT, 3AOT, and control conditions (2AOT: adjusted *p* < 0.05, *r* = 0.61; 3AOT: adjusted *p* < 0.01, *r* = 0.86; CON: adjusted *p* <.01, *r* = 0.87). A table showing the median, first quartile, third quartile, 95% confidence interval, test statistic (*χ*^2^(3) or *Z*), *p*-value, and effect size in all data comparisons is provided in Table S4.

### 3.4. Summary

Summarizing the comprehensive findings of this study, it was observed that the maximum number of rotations for the right hand (intervention hand) increased significantly across all conditions following the intervention, with the increase in the 2 min observation condition (2AOT) being notably greater than that in the control condition. For the left hand (non-intervention hand), significant increases were noted in the 1AOT, 2AOT, and 3AOT conditions, with changes in the 3AOT condition tending to exceed that of the control condition. Regarding errors, significant reductions were observed in the 1AOT, 3AOT, and control conditions for the right hand, although no significant differences were detected between the conditions. In the assessment of mirror neuron system activity (IMR), APB activity was significantly increased in the 2AOT condition, with this change being significantly greater than that in the control condition. Furthermore, compared with the 1AOT condition, the 2AOT and 3AOT conditions resulted in higher levels of mental fatigue, and the 3AOT condition exhibited lower concentrations. These findings indicate that the duration of observation exerts time-dependent differential effects on manual dexterity, mirror system responses in the brain, and subjective psychological burden.

## 4. Discussion

This study aimed to investigate the impact of different observation durations on manual dexterity, MNS activity, and subjective psychomotor effort. The primary results showed that 2 min of observation duration improved dexterity in the right hand (intervention hand) and increased MNS activity. In addition, dexterity of the left hand (non-intervention hand) improved after 3 min of observation. Furthermore, compared to the 1 min observation, the 2 min and 3 min observations resulted in higher mental fatigue, and the 3 min observation showed lower concentration levels.

### 4.1. Enhanced Manual Dexterity and Mirror Neuron Activity with 2 min Observation

Our findings demonstrated that 2 min of motor observation improved hand dexterity (the maximum number of rotations) and MNS activity in the intervened hand. A sub-analysis of a previous meta-analytic study [20] reported that observation durations longer than 3 min were more effective than observation durations shorter than 3 min in the effect of AOT on arm function. The discrepancy between these results and our current findings is presumably due to differences in the targeted body part and type of task. The body parts targeted in our investigation were the hand or fingers, and the task involved complex, skillful movements. A separate meta-analysis [11] reported that AOT was more effective in improving hand function than arm function. Therefore, it is reasonable to conclude that even observation periods shorter than 3 min can have beneficial effects on hand and finger functions.

Interestingly, in our study, both behavioral performance and MNS activation improved only at a specific time period of 2 min rather than at 1 or 3 min. Although numerous studies have examined mirror neuron system activation during AOT based on various task types [42] and parameters, few have investigated time- or quantity-dependent changes in MNS activity, leaving a significant knowledge gap in this area. To the best of our knowledge, only one previous investigation [43] has shown that MNS activity captured by EEG gradually increases from the initial phase over multiple AOT sessions. These findings suggest that MNS may exhibit plasticity in a time- or quantity-dependent manner. This quantity-dependent plasticity perspective helps explain the increased MNS activity and performance improvement observed with the 2 min observation compared to the 1 min observation in our study. However, this explanation alone does not account for why these improvements failed to persist or increase further during the 3 min observation period. Two alternative explanations account for this phenomenon. The first is the suppression of activity by repetitive stimulation, that is, neural adaptation. Functional brain imaging studies [44] have reported that the activity of MN cell populations is reduced by repetitive stimulation. Another possibility is the effect of observer attention or the cognitive load. Previous studies [45] have shown that the IMR during motor observation is reduced by decreased observer attention and increased cognitive load. This is supported by the results of the subjective psychomotor effort in the present study, which showed lower levels of concentration and greater mental fatigue during the 3 min observation period than during the 1 or 2 min observation periods. Therefore, it is possible that the IMR did not increase during the 3 min observation period due to the effects of neural adaptation or cognitive load from repetitive stimuli, beyond the dose-dependent plastic increase.

Regarding the number of errors during the ball rotation task, the intervention conditions were not significantly different from the control conditions in either hand. This result indicates that the variation in observation duration did not significantly impact the accuracy of performance and can be interpreted as indicating that there was no trade-off effect between rotation speed and accuracy in performance.

This study revealed minimal IMR changes in the ADM, potentially reflecting the muscle-specific nature of IMR, wherein activation predominantly corresponds to muscles directly engaged in the observed behavior [35,46]. Thumb-associated muscles likely contributed more substantially to hand movements during the ball rotation task than those associated with the little finger. Consequently, observation of ball rotation motion, primarily a thumb-dominant movement, appeared more effective at eliciting IMR in the APB, while demonstrating limited sensitivity in detecting ADM-related IMR changes.

### 4.2. Enhanced Manual Dexterity of the Non-Intervention Hand with 3 min Observation

The 3 min observation period in this study improved dexterity in the non-intervention hand. The phenomenon in which the learning effect of one limb is transferred to the contralateral limb is called “intermanual transfer” and has long been known as a motor learning effect based on actual movement [47]. The main neural bases of the bimanual transition include the primary motor area [48,49], supplementary motor area [49], premotor area [48,50], inferior parietal lobule [51,52], fronto-parietal network [50], dorsal attention network [50], and hippocampus [50], and these brain regions include the MNS [53]. Recent reports [28,29] indicate that the transition effect occurs not only during exercise execution but also during exercise observation, and the results of this study support this view.

Interestingly, however, in the present study, this transition effect occurred with an observation duration of 3 min, instead of 2 min. At first glance, this result seems to contradict the results of increased dexterity and MNS activity seen in the intervention hand with the 2 min observation. However, they can be interpreted from three viewpoints. The first is the relationship between the learning of the intervention hand and transfer learning of the opposite hand. Prior studies have shown that unilateral learning scores do not necessarily correlate with contralateral transition scores [50]. Therefore, it is possible that the learning effect of the dominant hand on the AOT intervention side in the current study may not necessarily translate to the non-dominant hand.

The second is the influence of the observer’s perspective on MNS activity. The videos used in this study were obtained from a first-person perspective. Observation of movement from a first-person perspective is more likely to activate the observer’s mirror neurons and motor-related areas than from a third-person perspective, improving performance of the same hand that is being observed in an effector-dependent manner [53,54].

The third is the effect of different types of intermanual transfer. In general, there are two types of transition effects: mirror (the same motion command) and non-mirror (the same visuospatial coordinates). In the case of the ball rotation task in this study, the transfer effect was a mirror type because the performance of the right hand evaluated the counterclockwise rotations and that of the left hand evaluated the clockwise rotation operation. According to previous studies [29], the transfer effect from action observation is smaller under mirror conditions than under non-mirror conditions. Therefore, it is conceivable that the transition effect of the mirror condition due to the motion observation was not strong, that the 2 min observation was not sufficient, and that it may have appeared at 3 min.

Summarizing these three viewpoints, the learning effects of the AOT intervention hand and the transfer effects of the non-intervention hand may involve different mechanisms. With 2 min of observation duration, first-person perspective observational learning strongly activated the MNS, producing effector-dependent effects on the intervention hand. In contrast, with three minutes of motor observation, after the initial formation of direct motor representations by the MNS, there was a transition to the formation of more abstract and effector-independent motor representations, which may have gradually promoted mirror-type transfer effects to the non-intervention hand over time.

### 4.3. Subjective Psychomotor Efforts (Concentration, Physical Fatigue, and Mental Fatigue)

Subjective psychomotor efforts showed that attention and cognitive load during the task differed depending on observation duration. There is also a trade-off relationship between psychomotor effort and the effect on hand dexterity or activation of the mirror neuron system. This suggests the importance of considering the balance between the observation duration and psychomotor effort in task settings to optimize the effects of AOT.

Contrary to expectations, the observation of still images of geometric patterns in the control condition resulted in a lower level of concentration and higher physical and mental load. As for the reason for this, compared to motion observation videos, still images may have been more easily boring because of their lack of movement. Additionally, geometric patterns have a high degree of visual heterogeneity as stimuli, potentially causing fatigue.

### 4.4. Limitations of This Research

In this study, the observation duration for each intervention condition, execution time, and number of interventions were controlled between conditions; however, the total AOT implementation time and the ratio of observation to execution time could not be controlled. Therefore, it remains unclear whether the results obtained are due to differences in the pure observation duration or variations in the total AOT implementation time or observation-to-execution ratio. Future research with experimental designs that control for these variables will be necessary.

This study provided basic knowledge limited to healthy young individuals. However, the effects of aging and stroke-induced brain damage have not yet been examined. Brain activity during action observation and execution differs between patients with stroke and healthy adults [55,56], and behavioral performance and psychomotor efforts during AOT are likely to differ. Multilateral assessment of the effects of observation duration on patients is needed, considering brain injury severity, hand motor dysfunction, and disease stage (recovery process and neuronal plasticity susceptibility). Furthermore, while the inclusion criterion for right-handed individuals was established to minimize the influence of handedness on MNS activity, this decision consequently restricts the generalizability of our findings to left-handed individuals. Additionally, the stringent exclusion criteria implemented for TMS safety considerations inevitably reduce the generalizability to potential populations meeting these criteria.

In this study, we employed still images as the control condition. However, it is important to acknowledge the potential for varying placebo effects between still image observation and video observation. Participants may anticipate more pronounced effects during video observation, and this possibility cannot be dismissed as a factor influencing the results. Notably, this variation in effect may complicate the interpretation of results when comparing the still image condition with each video observation condition (1 min, 2 min, and 3 min). Future research should consider implementing control conditions with more comparable placebo effects, such as observation of unrelated motor actions with similar visual complexity or videos showing objects moving without human agency. These alternatives would provide a more comparable visual stimulation while controlling for the specific action observation effects being studied.

A further consideration is that the specifics of “how” to observe during exercise observation may have influenced performance. As the present study only provided general verbal instructions to encourage careful observation, we may not have controlled for the more specific methods and strategies used by individual participants during movement observation. Prior research has indicated that MNS activity varies with differences in gaze eye movements during observation [57]. It is also possible that some participants imagined the movement during the observation and rehearsed for the subsequent execution of the movement. Previous studies have shown that individual differences in motor imagery ability are closely related to motor adaptive abilities [58]. Therefore, it would be desirable in future studies to instruct observation methods that explicitly refer to eye gaze and motor imagery strategies during exercise observation, and to simultaneously measure and evaluate these strategies to explore their relationship to performance.

Our error definition system was limited to ball contact and drops, which may not capture the full spectrum of performance errors. Future research should consider a more comprehensive error taxonomy including: rotation cessations or interruptions (when ball movement momentarily stops); hand posture deviations (when the palm significantly tilts from the horizontal plane); finger movement errors (e.g., uncoordinated finger movements, excessive force application, and inappropriate finger positioning); trajectory deviations (e.g., balls moving outside optimal rotation paths without falling); and momentary movement cessations. Such an expanded error classification would provide a more nuanced assessment of motor performance and potentially reveal subtle effects of different observation durations on specific aspects of motor execution.

Finally, this study evaluated only the immediate effect of a single AOT intervention, and not multiple-week interventions or long-term sustained effects relevant to clinical practice. To develop a more practical and effective treatment model, investigation of long-term effects is essential.

### 4.5. Clinical and Academic Significance

The present study focused on the most basic AOT methodology, observation duration, which is an important parameter thought to affect intervention effects and treatment continuity. Therefore, the findings of this study provide practical knowledge that can help achieve more efficient AOT in clinical settings. The study also examined the effects of different observation durations from three multifaceted aspects: behavioral, neurophysiological, and psychomotor. The insights provided on these interactions will contribute to the elucidation of the mechanism of action of AOT.

## 5. Conclusions

This study investigated how different observation durations affect manual dexterity, MNS activity, and subjective psychomotor effort. Our results showed that 2 min observation improved right hand (intervention hand) dexterity and increased MNS activity, while 3 min observation improved left hand (non-intervention hand) dexterity but led to higher mental fatigue and lower concentration compared to 1 min observation.

Limitations include our inability to control the total AOT implementation time or observation-to-execution ratio between conditions, variation in potential placebo effects between still image and video observation conditions, lack of control for specific observation strategies used by participants, limited error definition system that may not capture the full spectrum of performance errors, restriction to healthy young right-handed participants, and evaluation of only the immediate effects of a single intervention. These factors may limit the generalizability of our findings to clinical populations and left-handed individuals.

This study provides practical knowledge about observation duration, a fundamental AOT parameter, and offers insights into AOT mechanisms through behavioral, neurophysiological, and psychomotor assessments. Future research should investigate long-term effects in clinical populations considering factors such as brain injury severity and recovery stage, optimize observation–execution ratios, implement control conditions with comparable placebo effects, include more comprehensive error taxonomies, measure specific observation strategies, and correlate clinical improvements with neural network changes to better understand AOT’s neuroplastic processes.

## Data Availability

The original contributions of this study are included in the article/Supplementary Material. Further inquiries can be directed to the author (e-mail: esuzuki@yachts.ac.jp).

## Supplementary Materials

The following supporting information can be downloaded at https://www.mdpi.com/article/10.3390/brainsci15050457/s1: Table S1: The number of rotations during the ball rotation task; Table S2: The number of errors during the ball rotation task; Table S3: Interpersonal motor resonance (IMR); Table S4: Subjective psychomotor effort after AOT.

## Abbreviations

The following abbreviations are used in this manuscript:

ADM	Abductor digiti minimi
AOT	Action observation therapy
APB	Abductor pollicis brevis
IMR	Interpersonal motor resonance
MEP	Motor evoked potential
MN	Mirror neuron
MNS	Mirror neuron system
NRS	Numerical rating scale
RMT	Resting motor threshold
SPE	Subjective psychomotor effort
TMS	Transcranial magnetic stimulation
